# MeCP2 deficiency impairs motor cortical circuit flexibility associated with motor learning

**DOI:** 10.1186/s13041-022-00965-0

**Published:** 2022-09-05

**Authors:** Yuanlei Yue, Ryan T. Ash, Natalie Boyle, Anna Kinter, Yipeng Li, Chen Zeng, Hui Lu

**Affiliations:** 1 Department of Pharmacology and Physiology, School of Medicine and Health Sciences, The George Washington University, Washington, DC 20037 USA; 2 Department of Psychiatry, Stanford University, Palo Alto, CA 94305 USA; 3 Department of Physics, Columbian College of Arts and Sciences, The George Washington, University, Washington, DC 20037 USA

**Keywords:** MeCP2, Rett syndrome, Regression, Motor deficit, Motor cortex, Neural circuit, Synchronization

## Abstract

Loss of function mutations in the X-linked gene encoding methyl-CpG binding protein 2 (MECP2) cause Rett syndrome (RTT), a postnatal neurological disorder. The loss of motor function is an important clinical feature of RTT that manifests early during the course of the disease. RTT mouse models with mutations in the murine orthologous *Mecp2* gene replicate many human phenotypes, including progressive motor impairments. However, relatively little is known about the changes in circuit function during the progression of motor deficit in this model. As the motor cortex is the key node in the motor system for the control of voluntary movement, we measured firing activity in populations of motor cortical neurons during locomotion on a motorized wheel-treadmill. Different populations of neurons intermingled in the motor cortex signal different aspects of the locomotor state of the animal. The proportion of running selective neurons whose activity positively correlates with locomotion speed gradually decreases with weekly training in wild-type mice, but not in *Mecp2*-null mice. The fraction of rest-selective neurons whose activity negatively correlates with locomotion speed does not change with training in wild-type mice, but is higher and increases with the progression of locomotion deficit in mutant mice. The synchronization of population activity that occurs in WT mice with training did not occur in *Mecp2*-null mice, a phenotype most clear during locomotion and observable across all functional cell types. Our results could represent circuit-level biomarkers for motor regression in Rett syndrome.

## Introduction

Loss-of-function mutations in methyl-CpG-binding protein 2 (*MECP2*) cause Rett syndrome (RTT), a severe pediatric neurological disorder [1]. A distinguishing and sometimes tragic feature of RTT is the pronounced motor regression that occurs following some advancement through developmental milestones. Apraxias, stereotypies, aphasia, and impaired locomotion are all common in RTT.

Although the molecular lesions causing RTT are becoming better understood [2], how these molecular changes lead to progressive alteration of the behavior and associated neural circuit function remains unknown. Clinical studies in Rett patients suggest that motor cortical physiology may be particularly disrupted in Rett patients. For example, Rett patients have abnormal electroencephalogram (EEG) oscillations over frontal regions which correlate with stereotyped repetitive hand-wringing [3], and EEG recording during movements suggests abnormal cortical control of corticospinal neurons with intact corticospinal function [4]. Furthermore, neocortical neuron dendritic pathology is more pronounced in motor frontal regions compared to posterior areas in Rett brains postmortem [5].

Modeling of Rett syndrome in mice has granted a range of insights into the pathophysiology of motor phenotypes in this disorder. Both male *Mecp2*-null and female heterozygous Mecp2 ± mice show progressive motor impairment [6, 7]. Conditional knockout of MeCP2 from excitatory neurons leads to a much more pronounced motor phenotype than conditional knockout from inhibitory cells, in fact, worse than the constitutive Null, suggesting that excitatory neuronal function is especially important for motor dysfunction [8–10]. Morphological study showed that MeCP2 deficiency causes decreased spine density and disorganized axons in the motor cortex [11]. *Mecp2*-null mice have a pronounced decrease in local excitatory but not inhibitory connectivity in the motor cortex [12]. While using in utero transfection of short hairpin RNA constructs to knock down MeCP2 expression in a sparsely distributed subset of layer (L) 2/3 pyramidal neurons in the motor-frontal cortex of wild-type mice, it was found that MeCP2 deficiency leads to > 30% reduction in local excitatory input from middle cortical layers (L3/5A), but not inhibitory inputs, compared with MeCP2-replete controls [13]. These studies show that MeCP2 deficiency in cortical pyramidal neurons induces pathological synaptic defects in excitatory intracortical circuits in the motor cortex. Recently, we also found that the motor cortical circuit in MeCP2 deficient mice retains enough plasticity to support learning [14]. However, it remains unknown how these synaptic pathologies disrupt neuronal circuit activity leading to the progression of motor deficit in Mecp2 mutant mice.

The goal of this study was to explore the effect of Rett-associated neuropathological changes on the dynamics of motor cortex activity during movement. We focused on two core features of motor cortex neuronal activity: (1) different ensembles of motor cortical neurons are activated during different behaviors [15–17], and (2) with learning ensemble activity is gradually refined and subgroups of neurons become functionally synchronized to more reliably drive behavior [18–21]. We recorded from motor cortical pyramidal neurons in L2/3, the primary input to L5 corticospinal neurons projecting to the spinal cord to orchestrate movements [16, 22]. The activity was measured during induced locomotion on a motorized wheel-treadmill, a motor behavior that is associated with complex motor cortical activity [23, 24], but does not rely on reward-based learning which is impaired in *Mecp2*-mutant mice. We will measure whether MeCP2 deficiency alters the selectivity of motor cortical neurons for locomotor movements and the correlation of neuronal ensemble activity during locomotion. Furthermore, we will examine whether changes in M1 function relate to the disease progression in *Mecp2*-null mice. We found that the population code for locomotion speed refines over time, with progressively fewer running-selective neurons over weeks of training in WT mice. This refinement is lost in *Mecp2*-null mice. Speed-selective and rest-selective neurons are clustered in the motor cortex, but this functional organization is less pronounced in Null mice. In WT mice, a progressive synchronization of neuronal ensemble activity occurs over several weeks of training on the motorized locomotion wheel. This synchronization is absent in Mecp2 mutant mice. The loss of neuronal ensemble synchronization is most prominent in activity recorded during locomotion and occurs in rest-selective, running-selective, and non-selective cells. Our results provide a circuit-level biomarker for motor regression in this mouse model of RTT.

## Materials and methods

### Animals

Mice were housed in an AAALAC-certified Level 3 facility on a 14-h light cycle. Male *Camk2-cre; Mecp2*^*−/y*^ (Null) mice were obtained by breeding heterozygous female *Mecp2*^±^ mice on the 129S6SvEvTac strain (*1*) obtained from Dr. Huda Zoghbi to male *Camk2-cre* mice carrying *Camk2-cre* allele on the C57/B6 strain obtained from Jackson (JAX#005359).

All procedures to maintain and use these mice were approved by the Institutional Animal Care and Use Committee for the George Washington University.

### Surgeries

At the age of 3–4 weeks old, male mice were deep anesthetized and then locked onto the stereotaxic equipment. After prepping a 3 mm-wide craniotomy was drilled over the motor cortex using a high-performance surgical drill. The center of the craniotomy was ~ 1.6 mm lateral to bregma (*2*). AAV/DJ-flex-GCaMP6m virus was injected into the forelimb area of the right motor cortex (1.5 mm lateral and 0.3 mm anterior to bregma, according to previous studies [14] at depth of 250 μm via a 1.0 mm O.D. glass microneedle with a 10–20 µm diameter tip attached to a Nanoject microinjector pump. A glass coverslip was then placed over the exposed brain and sealed into the skull. The surgical site was then closed using vet glue. A 2-g titanium head post washer was attached to the head with "cold cure" denture material for later head-fixation under a two-photon microscope.

### Chronic in vivo two-photon imaging

Experimental animals were placed head-fixed on a wheel under light isoflurane sedation, then allowed to wake up. The mouse was acclimated to the wheel in a free running mode for 10 min, head-fixed under a Thorlabs Bergamo II two-photon microscope (Thorlabs, NJ) with a Nikon 25X (NA 1.1) water-immersion objective lens (Nikon Instruments Inc., NY). Time-lapse images were acquired with Thorlabs software at 6–7 frames/sec for 4630 frames for 12 min (2 min for each speed for both increasing and decreasing mode). The interval between two modes at each trial is 1 min. The laser power was adjusted to the minimum necessary to achieve ideal fluorescence intensities during each imaging session. On average, about 20–30 mW of power arrives at the sample. The scanning depth is ~ 200 µm from the dura. After completing the experiment, the animals were perfused with 10% formaldehyde, and the brain was sectioned to confirm the imaging depth and the location of the viral expression.

### Behavioral paradigm

**Fig. 1:**
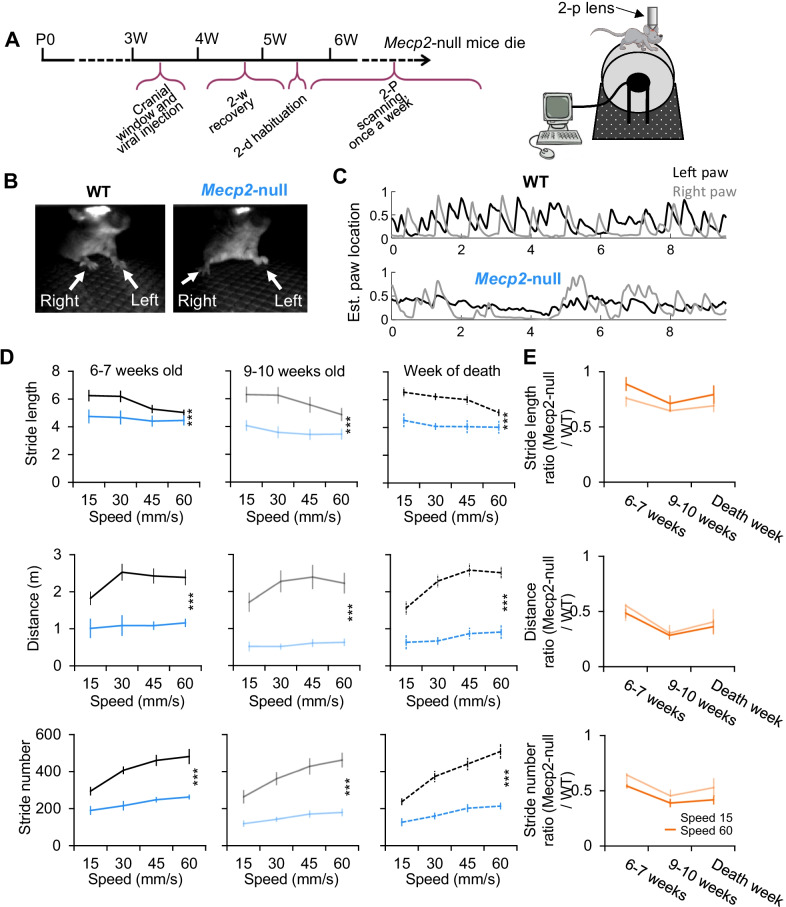
Chronic in vivo Ca2 + imaging on area M1 neurons of *Mecp2-null* (Null) mice and wild type (WT) littermates during rest and locomotion. **A** Left, Experimental time frame of chronic in vivo Ca2 + imaging. Right, Paradigm for studying locomotion in area M1. This involves accommodating the animals to run on a wheel that controlled by a motor which allows to set the speed of the wheel at variable levels (left) and monitor the neuronal activity of motor cortical neurons expressing GCaMP6m via viral infection (right) under two-photon microscope. **B** Capture of mouse locomoting on the treadmill during Ca2 + imaging under 2-photon microscope. The arrow heads point to the left and right paw, showing the alternative orientation of two paws from WT mice (top) and parallel orientation from *Mecp2*-null (mice) during locomotion. **C** Temporal trace of left and right paw position on the treadmill. X axis represents the time, and the Y axis represents the estimated paw location. The WT mouse makes large steps and mostly alternates between left and right forelimbs, while the *Mecp2*-null mouse’s movements are smaller and locomotion is irregular indicative of impaired motor coordination. **D** Averaged stride length (top), locomotion distance (middle) and stride number (bottom) at each speed at the age of 6 and 7 weeks, 9 and10 weeks and the week when the *Mecp2*-null mice died. Black, WT (n = 12); Blue, *Mecp2*-null (n = 15). F (1, 84) = 16.56; P = 0.0001 for Stride length of 6–7 weeks old; F (1, 68) = 42.50; P < 0.0001 for Stride length of 9–10 weeks old; F (1, 84) = 49.88; P < 0.0001 for Stride length of week of death; F (1, 84) = 70.96; P < 0.0001 for Distance of 6–7 weeks old; F (1, 68) = 106.6; P < 0.0001 for Distance of 9–10 weeks old; F (1, 84) = 164.5; P < 0.0001 for Distance of week of death; F (1, 84) = 124.0; P < 0.0001 for Stride number of 6–7 weeks old; F (1, 68) = 121.4; P < 0.0001 for Stride number of 9–10 weeks old; F (1, 84) = 130.5; P < 0.0001 for Stride number of week of death; P values are from the main effects of Two-way ANNOWA test. **E** Ratio of averaged stride length (top), locomotion distance (middle) and stride number (bottom) between *Mecp2*-null and WT groups at speed 60 mm/sec at the age of 6–7 weeks, 9–10 weeks and the week when the *Mecp2*-null mice died, showing the regression of locomotion behavior

Three weeks after the surgery, experimental animals were trained on a computerized wheel treadmill system customized by Delta Commercial Vision (NJ, USA) while two-photon imaging was performed simultaneously. A motor that drives the running of a foam wheel with 6 inches in diameter was controlled by an Adreno chip connected to a computer via a USB cable. Before the start of each trial, the experimental animal was put on the treadmill with head fixed at a comfortable height under the two-photon microscope. ThorCam software controls the videotaping of the mouse behavior at 71.5 Hz via a high-speed camera (Thorlabs, NJ) and simultaneously triggers the ThorImage software (Thorlabs, NJ) to start two-photon imaging and the digital command that controls the treadmill. The experimental animals learned to adapt to induced running on the treadmill at different speeds achieved by the electrical power supplied to the motor at different levels encoded by the digital command. The trial is composed of three sessions: (i) session 1 is 12 min in total. It starts at speed 0 (rest) and increases by 15 mm/sec every 120 s up to 60 mm/sec, followed by a 2-min rest period. (ii) Session 2 is a 1-min interval for data saving and the rest of the animal. (iii) Session 3 starts at speed 0 followed by 60 mm/sec and decreasing by 15 mm/sec every 120 s down to 0 mm/sec. There are five total speeds: 0, 15, 30, 45, 60 mm/sec, 2 min per speed. This experiment was performed weekly (one trial per week, Fig. 1A).

### Data analysis

Raw calcium imaging movies were imported into Matlab and motion-corrected with a published algorithm [25]. A previously-published cell-detection, neuropil-demixing, and calcium deconvolution algorithm was applied to the data [26]. For functional cell subtyping and event rate analysis, a minimum event threshold of 20 was applied to the deconvolved estimated firing rate. Event rate was calculated in individual bins as the sum of frames above the threshold within a 200frame bin, and then averaged across the 10–15 bins within each locomotion speed.

For event synchrony analysis, slow fluctuations in calcium were excluded using an 11-frame van Herk minimum filter (dividing each value by the lowest value within 11 frames of that value) on the deconvolved trace. To filter out low–amplitude fluctuations in the signal, a minimum event threshold of 100 was used, and frames below this threshold were set to 0. Event synchrony was calculated using the Pearson correlation on the deconvolved, thresholded trace of each pair of neurons within a movie. The first 40 frames (~ 5.5 s) of each movie was excluded to avoid any synchrony from neuronal responses to the start of imaging. The locomotion synchrony analysis was performed on the activity pooled across the intermediate (3 and 4.5 cm/sec) speeds. The distance between neurons was calculated using the ROI centers as x,y coordinates.

### Tuning fit algorithm

To measure the degree of neuron tuning in each cell, Poisson regression was used to regress the number of events against a tuning response function based on the rotation speed of the wheel. If the coefficient for this regression was large, indicating a significant relationship between the number of events and the rotation speed of the wheel, we identified the neuron as 'tuned' to a particular behavior. More precisely, each deconvolved trace was divided into non-overlapping bins of 200 frames (corresponding to approximately 30 s) and the number of events in each bin was counted (a threshold of T was used to identify events). The wheel speed for each bin was also recorded and used to construct a 'speed curve', based on a set of pre-specified 'templates.' For example, for the 'running selective' template, the speed curve would be 1 if the wheel was turning and 0 otherwise. The event counts, which are proportional to the event rate, were then regressed against the speed curve using a Poisson (or log-linear) regression model. The intercept term of the regression gives a measure of the neuron's overall firing rate, while the regression coefficient of the tuning function gives an estimate of how significantly the firing rate changes as a function of the wheel speed. For example, consider a recording with 4 bins with 4, 4, 10, and 10 events respectively, and with the wheel paused for the first two bins and in motion for the second two. For the 'running selective' template, we would perform a Poisson regression with the event counts as the response against the speed function 0, 0, 1, 1 as the predictor. The intercept term is approximately 1.4 (log 4) while the speed function regression coefficient is approximately 0.92 (log 2.5), indicating that the neuron was 2.5 times more active while the mouse is running. Note that the use of an intercept term controls for differences in the average firing rate so that our analysis does not falsely identify neurons with a high overall constant firing rate as being tuned to a particular behavior simply because of the large number of events in certain bins. Neurons with regression coefficients greater than a fixed threshold C (equivalently, with p-values below a fixed threshold) were identified as 'tuned' to the template.

The algorithm was applied taking into account 4 key parameters: calcium deconvolution, minimum event threshold, use of event amplitude (i.e. to count all events above the minimum threshold equally, or to count larger events as more activity than smaller events), and the bin duration for calculating event rate (e.g. eight 250 frame bins, ten 200 frame bins, twenty 100 frame bins). We found that our fitting results were consistent over varying combinations of each of these parameters, and we decided to (1) use the deconvolved trace, (2) set the minimum event threshold to 20 (in the arbitrary units of the Pnevmatikakis algorithm), (3) count all events above the threshold equally, and 4) to calculate event rates in 200 frame bins as these consistently generated tuning curves with small error bars that were consistent even when the data was split in half.

### Statistics

**Fig. 2:**
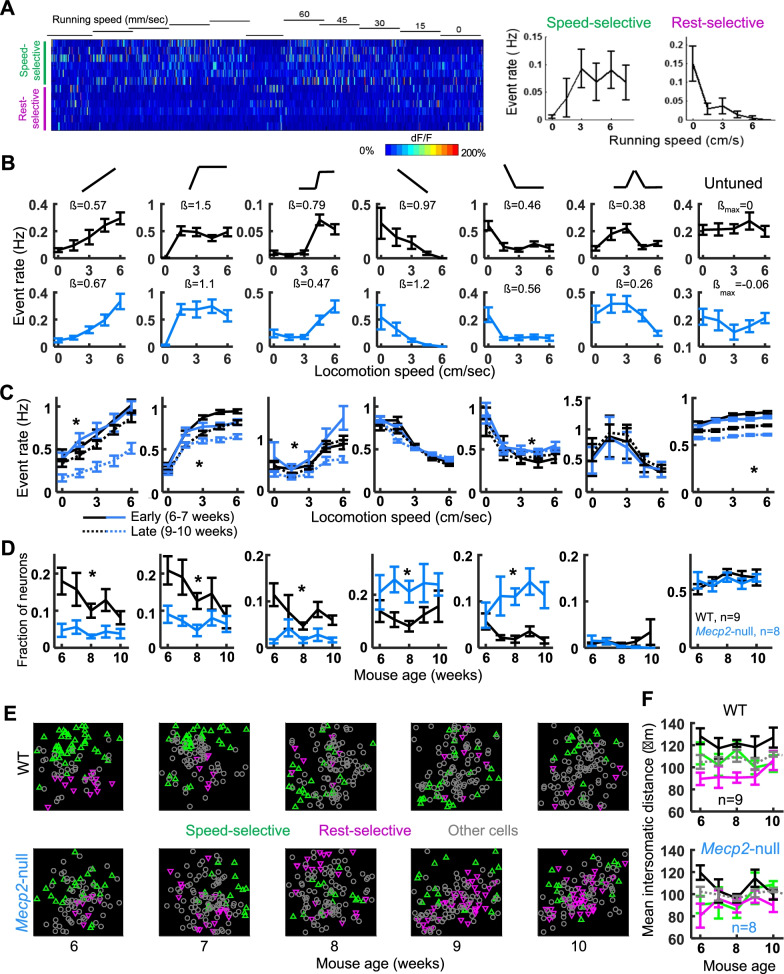
M1 neurons are tuned with respect to locomotion speed, and the distribution of tuning functions differs between the WT and *Mecp2*-null mice when motor deficit progresses. **A** Left, heat map plot of Ca2 + activities showing two types of neurons: speed-selective and rest selective. Each row represents one neuron. The bar under the speed-indicating numbers represents 150 s-long. Right, relation between activity and running speed of speed-selective and rest-selective neurons. **B** Example event rate by locomotion speed tuning curves for each cell type and genotype. β value represents the coefficient of Poisson regression which was used to regress the number of events against a tuning response function based on the rotation speed of the wheel. The larger the value, the more significant relationship between the number of events and the rotation speed of the wheel. **C** Mean tuning curves for each cell type and genotype, for early (6–7 weeks, solid lines) and late (9–10 weeks, dashed lines) imaging sessions. **D** Proportion of neurons that are significantly tuned to each tuning curve shape across mouse age for *Mecp2*-null (blue, n = 8) and WT (black, n = 9) mice. *, *P* < 0.01, genotype by time interaction, mixed effects models ANOVA. **E** Example imaging ROIs showing physical locations of somata for speed-selective (green triangles), rest-selective (magenta triangles), and non-selective (gray circles) cells across imaging days. **F** Mean intersomatic distances between speed-selective cells (green line), between rest-selective cells ( magenta line), between speed- and rest-selective cells (black line), and between all cells (gray dotted line) across imaging days, for WT (top) and *Mecp2*-null (bottom)

**Fig. 3:**
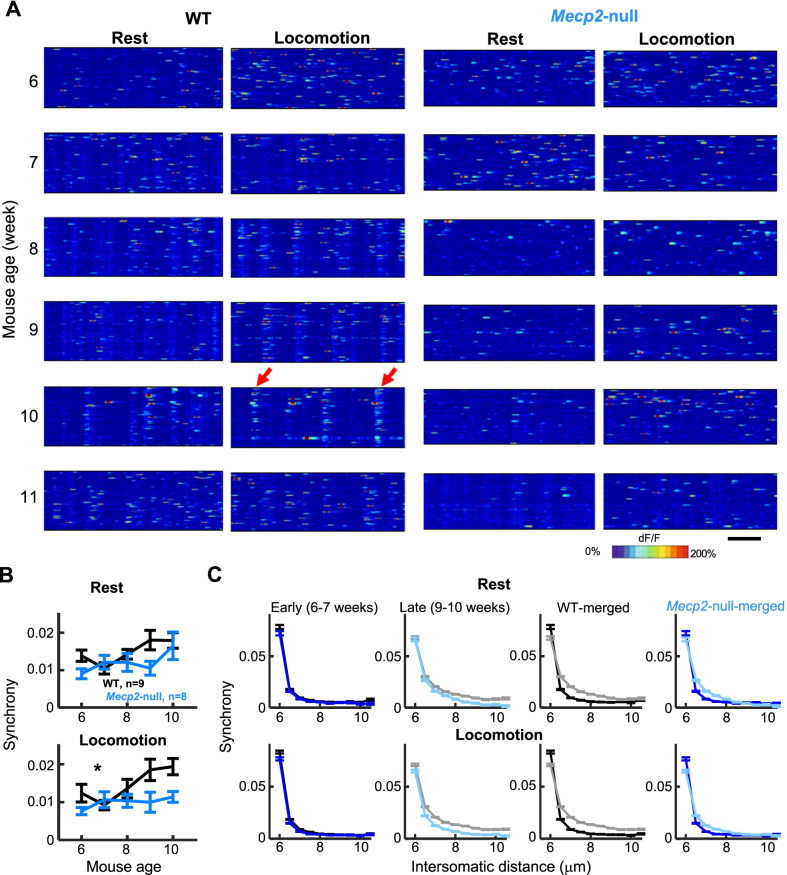
Synchrony of neuronal activities in area M1 changes differently between *Mecp2*-null mice and WT littermates when motor deficit progresses. **A** Heat map plot of Ca2 + activities from each week of a representative WT and *Mecp2*-null mouse showing synchrony changes during rest and locomotion over development. The bar represents 120 sec-long. **B** Averaged Pearson correlation coefficient across imaged ages during rest (left) and during locomotion (right). Black, WT (n = 9); Blue, *Mecp2*-null (n = 8). *P < 0.01, genotype by time interaction, mixed effects models ANOVA. **C** Event synchrony binned by intersomatic distance for early imaging sessions (weeks 6–7, 1st panel), late imaging sessions (weeks 9–10, 2nd panel), and early vs. late sessions for WT (3rd panel) and *Mecp2*-null (4^th^ panel)

**Fig. 4:**
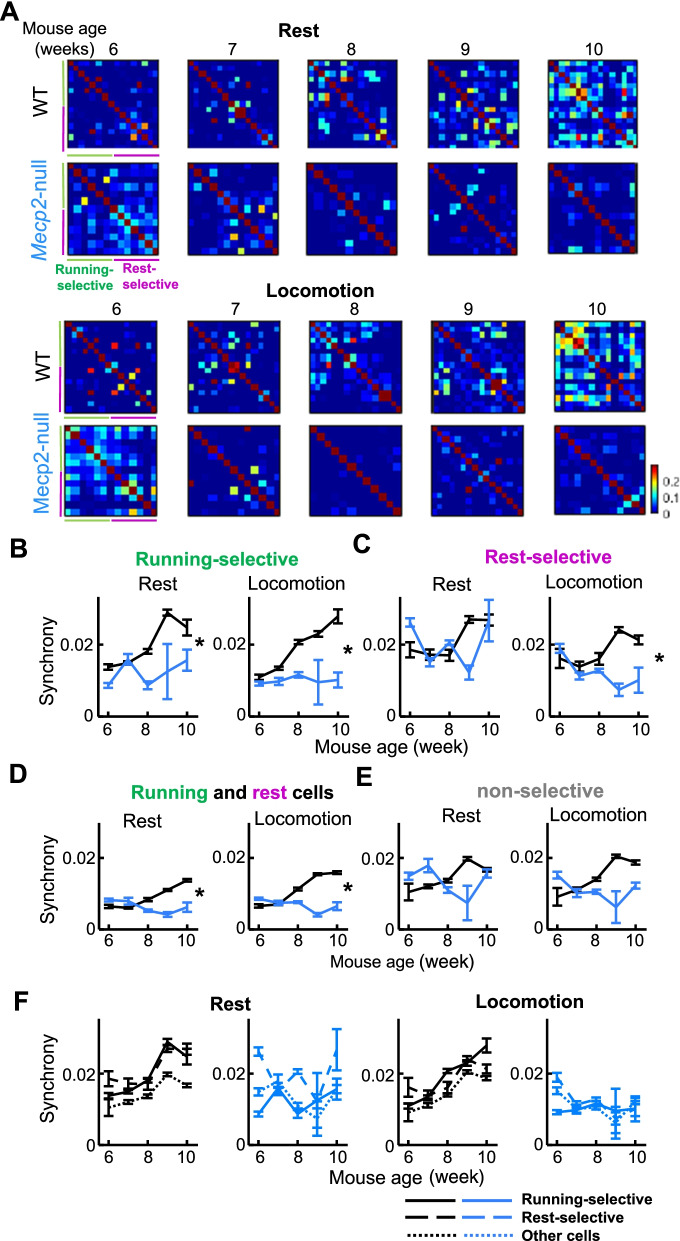
Neuronal synchrony in neurons differently tuned to locomotion speed. **A** Example correlation matrices sorted by cell type for a WT mouse and *Mecp2*-null mouse during rest (top) and during locomotion (bottom) across ages. The top 8 rows of each matrix are from running-selective cells, and the bottom 8 rows are from rest-selective cells. The top-left quadrant of each matrix shows Pearson correlations between running-selective cells, the bottom-right quadrant shows correlations for rest-selective, and bottom-left and top-right quadrants show correlations between cell types. **B**–**E** Synchrony within running-selective cells (**B**), rest-selective cells (**C**), between running-selective and rest-selective cells (**D**) and within non-selective cells (**E**) across ages, during rest (left) and locomotion (right). Black line, WT, Blue line, *Mecp2*-null. Error bars are the 95% confidence intervals calculated by subsampling pairs from each animal per imaging time point, the number of pairs set to guarantee the same numbers of pairs are analyzed from at least 5 animals per timepoint, and calculating the mean values 1000 times. *P < 0.05, genotype by time interaction, mixed effects models ANOVA. **F** Same data plotted in **D**–**E**, but with 3 different cell types plotted on the same graph, to show the differences between cell types. Left panels rest, right panels locomotion

Data were expressed as mean ± SEM. Sample sizes are based on the publications in the field and our preliminary experiments. Data were tested for normality using Shapiro–Wilk test and equal variance with Kolmogorov–Smirnov. Two-way ANOVA was used followed by Tukey’s post hoc test for multiple group comparison in Fig. 1 using GraphPad Prism 9 (GraphPad Software Inc., San Diego, CA, USA). Linear mixed models were used to obtain *P* values in Figs. 2, 3, and 4, as previously described [14].

## Results

### Area M1 neurons are tuned with respect to locomotion speed

Female heterozygous *Mecp2*^+/-^ female mice in 129-background were crossed to wild-type (WT) *Camk2-Cre* homozygous male mice in B6/C57 background. At 3 weeks of age (Fig. 1A), male *Mecp2*-null mice and WT littermates (F1 with C57&129 mixed genetic background) were implanted with a 3 mm cranial window over the anterior motor cortex (coordinates 1.6 mm lateral, 0.3 mm anterior to bregma) after AAV-flex-GCaMP6m was injected. Three weeks following the surgery (6 weeks of age), animals were acclimated to a head-fixed motorized foam wheel that forced the mice to locomote at five different speeds (rest, 15, 30, 45, and 60 mm/sec) (Fig. 1A). The training paradigm started with the rest condition and then the wheel speed increased stepwise, then another rest condition, followed by a trial starting at the top speed and decreasing stepwise (sequence: 0, 15, 30, 45, 60, 0, 60, 45, 30, 15, 0 mm/sec), as previously described [14]. Each condition lasted for 120 s. Initial training was performed daily for two days, followed by weekly chronic imaging over the same area (Fig. 1A, left). The mouse was placed under the two-photon microscope on an identical motorized foam wheel (Fig. 1A, right). The rotating dowel rod task was performed once per week to track the locomotion ability of imaged animals with disease progression. Data was acquired from 8 *Mecp2*-null and 9 WT littermate mice. The paw movement is automatically tracked by an algorithm [27]. Both front paws showed similar movements (Fig. 1B, C). Both WT and *Mecp2*-null mice performed successful locomotor paw movements on the motorized wheel. As observed by weekly testing on the wheel treadmill, *Mecp2*-null mice showed apparent motor deficit, reflected by shorter stride length and distance, as well as much lower stride numbers at each speed (Fig. 1D). Such motor deficit progressively worsened when the mice grow older (Fig. 1E). Data shown were analyzed from 6 weeks until the age of 10 weeks, as after that, half of the *Mecp2*-null mice were dead (and all were dead by 15 weeks of age).

### Motor cortical neurons in *Mecp2*-null mice showed weakened response to locomotion speed

While the mice were performing the locomotion task on the treadmill, we monitored the neuronal activity of L2/3 pyramidal neurons in the M1 using a 2-photon microscope. A single plane of GCaMP-labeled L2/3 pyramidal cells in the anterior motor cortex (6 Hz, 512 × 512 resolution raster scan, 1 µm per pixel, 10–20 mW laser power) was selected by two-photon imaging (generally ~ 200 microns cortical depth). Mice were imaged once a week in the same area (but not the same group of neurons) until the window lost clarity, or the animal died (in the case of *Mecp2*-null mice). Diverse patterns of activity were observed in motor cortical neurons both during rest and locomotion (Fig. 2A). An automated cell detection, neuropil-demixing, and calcium deconvolution algorithm was used to generate cellular calcium traces [26]. Fifty-nine to 136 (mean 110, S.D. 17) neurons were analyzed in each animal at each time point. Interestingly, the firing rate of some neurons was related to the animal’s locomotion; one subpopulation of neurons demonstrated firing that correlated positively with speed (Fig. 2A, running-selective cells); another population was selective to the rest conditions (Fig. 2A, rest-selective cells).

To analyze the encoding of locomotion speed across the population of L2/3 motor cortical neurons, a custom fitting algorithm was produced that fit each cell’s activity to six different shapes (Fig. 2B): Speed-correlated (linear positive relationship to speed), running-specific (most active during locomotion conditions), fast-tuned (active selectively at high running speeds), speed anti-correlated (linear negative relationship to speed), rest-specific (most active during rest conditions), and slow-tuned (active selectively at intermediate speeds). Example event rate by locomotion tuning curves for each cell type from each genotype are shown in Fig. 2B with their respective fit values. Notably, cells with fit betas above 0.2 clearly resemble the respective fit type. Thus, this index was used as the minimum to classify cells (results were similar for other minimum fit betas). Neurons were allowed to be tuned to multiple fit types (i,e, we did not arbitrarily classify neurons to a single fit type). To be counted as a significant fit, the cell had to demonstrate a good fit (beta > 0.2) in both the first half (i.e. with the speed increasing stepwise) and the second half (with the speed decreasing stepwise) of the data.

The mean event rate by locomotion speed is plotted for each cell type, pooled into early (6–7 weeks of age, solid line) and late (9–10 weeks, dotted line) imaging sessions in Fig. 2C. Interestingly, there was a prominent decrease in the event rate of speed-correlated cells in *Mecp2*-null mice at later ages that was not observed in the WT mice (Fig. 2C, 1st panel). Running-specific (2nd panel), fast-tuned (3rd panel), and untuned cells (7th panel) were also less active in *Mecp2*-null mice compared to WT at the later ages. Rest-selective cells (5th panel) had similar or slightly increased firing in mutants compared to WT.

### Loss of refinement in the population code for locomotion over time in *Mecp2*-null mice

The fraction of neurons with different locomotion tuning shapes was calculated per mouse per week, averaged within genotype, and plotted across weeks in Fig. 2E (blue, *Mecp2*-null, black, WT). The most prominent phenotype observable in the WT data was a gradual decrease in the fraction of running-selective cells (Fig. 2D, left 3 panels). This likely reflects the refinement of motor cortical ensemble activity known to occur with repeated training on a motor task [20, 28]. Rest-selective cells remained mostly stable over the same time frame. (Fig. 2D, fourth, fifth, and sixth panels), while the proportion of untuned cells slightly increased (Fig. 2D, right panel).

This iterative refinement of locomotion-related activity was not observed in Rett mice. The fraction of cells correlated to locomotion speed started off significantly lower in Rett mice and stayed low across training (Fig. 2D left 3 panels). The fraction of cells selectively active at rest, in contrast, was more common in the Rett mice (Fig. 2D, 4th and 5th panels), and this effect became more prominent with disease progression. Small fractions of neurons (~ 0.05–0.1) fired specifically at slow speeds (Fig. 2D, 6th panel), and this was not significantly different between genotypes. Taken together these results indicate that (1) neurons in the motor cortex encode different aspects of induced locomotion, (2) the proportion of neurons whose activity encodes locomotion speed decreases with task learning in WT mice, (3) this profile is lost in *Mecp2*-null mice, and (4) The proportion of neurons active during the rest condition increases with disease progression in *Mecp2*-null mice.

While there was considerable variability in the proportion of running-selective and rest-selective cells from mouse to mouse, the proportions were relatively consistent across time within individual mice, suggesting that the proportion depends on where precisely in the motor homunculus that was imaged [29]. We wondered if there also could be a fine-scale clustering of running-and rest-selective cells, similar to the previous observation that mouse M1 neurons that drive the same behavior are functionally clustered [17]. We therefore analyzed the relative physical locations of running-selective and rest-selective cells in our data set (Fig. 2E). Interestingly, we found that within-subtype intersomatic distances were significantly lower than across-subtype intersomatic distances (Fig. 2E, F). This was most prominent for rest-selective cells. Running-selective-to-Rest-selective intersomatic distances were slightly higher on average than the distance between all cells, suggesting that there is some repulsion between these cell types. While the within-subtype spatial clustering was mostly consistent across ages in WT (Fig. 2F top panel), this organization was lost after 6 weeks of age in *Mecp2*-null mice (Fig. 2F bottom panel).

### Loss of motor cortical neuron ensemble synchronization with learning in *Mecp2*-null mice

We next looked for temporal patterns in the motor cortex ensemble activity during rest and locomotion. A clear feature observable in the data from WT mice was the emergence of synchronous co-activation of subpopulations of neurons starting on the 3rd–4th week of imaging (Fig. 3A left columns). Synchronous activity during locomotion, quantified using the Pearson correlation on deconvolved, thresholded calcium traces, increased from 7 to 10 weeks of age in WT (Fig. 3A left panels, Fig. 3B black lines) [30]. Sorting the correlations by distance (Fig. 3C) showed that the increase in synchrony with training was specific to cells that were greater than 25 microns apart; cells closer than this actually decreased their synchrony with training. This synchronization is similar to the synchronization of motor cortical neuronal ensemble activity known to occur with motor memory consolidation [19]. The events were too rare to be involved in triggering individual steps, and qualitative analysis of the animal’s behavior in relation to synchronous events (e.g. foot slips) did not show any clear correspondence, favoring the possibility that it reflects an internal phenomenon like memory consolidation rather than a motor command.

Interestingly, in Rett mice, synchronous events were not observed at any age (Fig. 3A, right panels), and synchrony did not increase over time (Fig. 3B blue lines). The lower synchrony compared to WT was observable across all intersomatic distances (Fig. 3C). This phenotype could possibly reflect a loss of circuit consolidation in the motor cortex. We next analyzed the synchrony of the different functional subtypes of neurons (Fig. 4). Correlation matrices were visualized sorted by running-selective (green) and rest-selective (magenta) neurons for WT (top) and *Mecp2*-null (bottom), for rest (left) and locomotion (right), across imaging weeks (Fig. 4A). Correlations were higher within cell type (Fig. 4A top-left quadrant of each matrix for running-selective, bottom-right quadrant for rest-selective) than between cell types (bottom-left and top-right quadrants). Synchrony increased over time in the WT but not the *Mecp2*-null (Fig. 4A top vs bottom panels, Fig. 4B–F), an effect that occurred across all cell types and was most clearly seen during locomotion. This loss of synchronization between behaviorally-relevant neurons with learning could contribute to the motor regression observed in these animals.

## Discussion

This study measured the functional properties of motor cortex neurons during locomotion in WT mice and *Mecp2*-null mice. We found that there are neurons that selectively response to running or rest, respectively, intermingled in L2/3 of the motor cortex with some clustering in WT mice but not in *Mecp2*-null mice. Rest-selective cells in contrast increased over time, and the emergence of these rest-selective cells happened most prominently in the weeks before the animal expired, suggesting that their activity could relate to the animal’s disease progression. Furthermore, the synchronization of activity observed with training in WT mice was not observed in *Mecp2*-null mice, providing novel in vivo evidence for abnormal motor circuit function with MeCP2 deficiency.

The population code for induced locomotion speed in mouse motor cortex has not been previously measured before to our knowledge, but agrees with the known role of mouse L2/3 in encoding different task variables [15, 20]. The functional clustering of rest-selective and running-selective cells is similar to what was previously observed when comparing activity during free running versus grooming [17]. The variability in the proportion of rest-selective and running-selective neurons from mouse to mouse along with consistency within animals suggests that there is a cortical map for different behaviors, that likely aligns with the motor homunculus [29].

Reorganization of motor cortical neuron synaptic connections is a hallmark of motor learning [31, 32] and is believed to underlie the modification of neuronal firing specificity with training [21]. Excitatory synaptic connectivity and plasticity are profoundly disrupted in *Mecp2*-null mice [33], which could contribute to our observation that the *Mecp2*-null motor cortex is unable to refine its firing selectivity over time. The synchronous events that we observed in WT synchronous events occurred at an order of magnitude slower timescale than locomotion, so we doubt that these ensemble events drive behavior directly. It is tempting to speculate that these events could, perhaps similar to the synchronous bursts observed during slow-wave sleep in the hippocampus, provide an excitatory drive for associative synaptic potentiation and thereby represent a circuit-level mechanism for motor memory consolidation. As L2/3 neurons in the motor cortex are considered to generate activity patterns that help the corticospinal neurons in the deeper layers to produce correct outputs for movement control, the lack of change in synchrony between L2/3 motor cortical neurons in *Mecp2*-null mice may impair the information processing within the motor circuit, leading to the loss motor skill learning. Therefore, loss of circuit consolidation in the motor system might underlie apraxia in Rett patients; loss of this mechanism in other areas could contribute to the global intellectual disability and impaired language seen in patients.

More interestingly, our recent study showed that *daily* training in the 8–10-week-old Mecp2-null mice can lead to improvement in motor coordination and alteration in synchrony between L2/3 neuronal pairs [14]. In contrast, *weekly* training failed to show such an impact. It highlighted that training intensity matters during the regression of motor function in Mecp2-null mice. This might be the case for the mouse models of many other neurological disorders with symptomatic motor regression.

## Data Availability

All data needed to evaluate the conclusions in the paper are present in the paper. The datasets used and/or analyzed during the current study are available from the corresponding author on reasonable request.

## Abbreviations

MeCP2: Methyl-CpG-binding protein 2
RTT: Rett Syndrome
EEG: Electroencephalogram
L2/3: Layer 2/3
Camk2: Ca2 + /calmodulin-dependent protein kinase 2
